# On Surgical Uses of the Strong Elastic Bandage Other Than Hæmostatic1.The bandage I use resembles Esmarch’s. I, however, call it the strong elastic bandage, for I used it continually very many years before the discovery which has immortalized an already illustrious name. I did this without the slightest notion that by such a bandage the deep arterial circulation of a limb could be entirely controlled, indeed with a very decided theoretical conviction that it could *not*, and therefore have no claim whatever to Esmarch’s discovery. Many years ago I twice re commended the elastic bandage to surgeons about to amputate legs, as a substitute for the common roller bandage often used to expel blood from a limb *before* and so save it for the patient *after* amputation, but my suggestion was disregarded. That is the nearest I ever came to making Esmarch’s great discovery.

**Published:** 1877-11

**Authors:** Henry A. Martin

**Affiliations:** Bv’t Lt. Col. and Late Surgeon U. S. Vols.


### ON SURGICAL USES OF THE STRONG ELASTIC BANDAGE[1] OTHER THAN HÆMOSTATIC.

By Henry A. Martin, M. D., Bv't Lt. Col. and Late Surgeon U. S. Vols.

At the meeting of the American Medical Association in your city last June, I made some extemporaneous remarks on the above subject. At the request of the Committee on Publication I have prepared a written abstract of those remarks, which may appear in the forthcoming volume of the Transactions. I have, however, been so often urged to publish a paper on the same subject for the immediate information of the profession, and the interest which has been manifested both at the meeting and since that time in numerous letters has been so unmistakable, that I gladly acquiesce, and offer the paper for your acceptance as a very slight acknowledgment of courtesy received individually at your hands, and generally at those of the whole profession and people of Chicago.

For over twenty-five years I have made use of a strong bandage of India rubber, for the treatment and cure of all ulcers of the lower extremity of a non-specific character,

1. The bandage I use resembles Esmarch's. I, however, call it the strong elastic bandage, for I used it continually very many years before the discovery which has immortalized an already illustrious name. I did this without the slightest notion that by such a bandage the deep arterial circulation of a limb could be entirely controlled, indeed with a very decided theoretical conviction that it could *not*, and therefore have no claim whatever to Esmarch's discovery. Many years ago I twice recommended the elastic bandage to surgeons about to amputate legs, as a substitute for the common roller bandage often used to expel blood from a limb *before* and so save it for the patient *after* amputation, but my suggestion was disregarded. That is the nearest I ever came to making Esmarch's great discovery.

coming at all within the category of curable, and as a most efficient aid to treatment and palliation in those of a specific character and those incapable of perfect cure by any method of treatment; and I may here say that a very large proportion of ulcers of the lower extremities practically incurable by other methods are capable of easy and permanent cure by this.[1]

The bandage which I use in the treatment of ulcers of the leg, is made of what is technically called "pure rubber," i. e., the best "Para" rubber, combined with the smallest possible mixture of sulphur, and subjected to the minimum of heat necessary to "cure" the gum and ensure it from the destructive changes which rapidly take place in bandages made of

---

1. I use such a bandage in the treatment palliative and curative of many other diseases and injuries, principally of the lower extremities; but of these I will, at the present time, give only a partial list, reserving a detailed account of its application to such cases for a future paper. The present writing will refer exclusively to the treatment of ulcers of the lower extremity. The principal cases, other than these, in which I have found such bandages eminently useful are, (and I mention them in the order of their importance, and the perfect applicability of this method to their treatment); (1.) Acute and chronic synovitis and consequent effusion in the joints, particularly of the knee, ankle, and elbow. (2.) Subluxations of those joints to which the bandage can be applied, both in their acute and chronic stages. (3.) Morbid effusion in the bursa mucosa, especially, after evacuation by aspiration, of the bursa developed over the patellæ, and known as "housemaid's-knee," and by other names. (4.) Œdema and anasarca, whether due to local or general causes, and occurring in either the lower or upper extremity, but chiefly, of course, the former. (5.) As an admirable palliative, and even in some instances, to a certain degree, means of curing varicose veins of the lower extremity, occurring either with or without ulcers. (6.) As a most efficient adjunct to treatment of erysipelas of either the legs or arms, whether traumatic or idiopathic, and, in very many cases, capable, without other means, of entirely curing, and, as it were, *extinguishing* that so called "*ignis* sancti Antonii." (7.) As a very valuable adjunct to the treatment of many cutaneous diseases, particularly when affecting the lower extremity, and as, without any other means whatever, local or general, absolutely and completely curative of many of these affections and their consequences. (8.) As a very useful surgical dressing after dislocation of any joint to which it can be applied, but particularly those of the knee elbow and ankle. (9.) As an extremely useful surgical dressing for some cases of that form of lesion entitled "green-stick-fracture," in which a pure uncured caoutchouc. The bandage is ten and a half ($10\frac{1}{2}$) feet in length ; three (3) inches wide, and of the thickness of no. twenty-one (21) of Stub's wire gauge. In about two inches of one end a piece of strong linen cloth is inserted. To this is strongly sewed a stout double tape of eighteen (18) inches in length. It is important that the edges of the bandages should be perfectly even, and this can only be accomplished by cutting them by machinery. The bandages I now use, are, with the single exception of attaching the tapes, prepared at the factory in which the sheet rubber is manufactured. In my first experiments, I attempted to make the bandages by cutting them, with strong, sharp shears, from the rubber sheet; but in this way it was impossible to produce

---

gradual, continually exercised, gentle pressure accomplishes that perfect "reduction" which cannot always be accomplished at once even by painful and unduly violent manipulation. Also in cases of deformity of bones from improper co-aptation of fracture, where the "callus" is too firm to admit of immediate bending, this sort of bandage, properly applied, has been found quite capable, by the constant, steady pressure which it exercises, of correcting deformity in fractured bones, even so long after injury as to be generally supposed capable of such correction by no means save that of re-fracture. (10.) As a most useful appliance eminently palliative, and sometimes even to a great degree rapidly and completely curative of injury of the ligamentous and other tissues of joints, resulting from contusion or other injury, or from relaxation of ligaments from disease. (11.) As a most efficient palliative of, an d often decided means of remedying chronic and acute inflammation of and about the joints or other parts of a limb; in phlebitis; as a preventive when abscess is threatened, or, in such cases, when too far advanced for prevention, as a means to hasten forward the process of suppuration. This enumeration of cases in which the strong elastic bandage has been found, *in actual practice*, very decidedly useful, although somewhat extended is, by no means, exhaustive. When the intelligent practitioner becomes familiar with, and reflects upon, the phenomena observed after the bandage has been applied; the effect of continuous, gentle, equable pressure, of perfect exclusion from air and light, of the constant moisture and equal warmth of the part involved in the bandage, of the constant support afforded to distended and weakened vessels, the relief of congestions by the mechanical expulsion of blood from the overloaded veins and capillaries, he will have little difficulty in perceiving the value of this method of treatment in the cases I have mentioned, and many others. I shall, at some future time, write a paper or two based on my personal experience in the use of the bandage for other cases besides those of ulcers of the lower extremity. At present I only wish to intimate what some of these cases are.

a bandage that would wear for any great length of time If there is the slightest notch in the edge, at that point, sooner or later, generally very soon, the bandage will tear, and become useless, while the machine-cut, perfectly even-edged bandage will bear continued and indefinitely repeated traction without any danger of such an accident. It is really astonishing how long such a bandage will wear. Many of my patients are wearing those, which have been in constant daily use for two, three, and even four years ; and I have cured several successive poor patients with bandages which still remain serviceable. The length and width stated are those I have found suited to the vast majority of cases. In a few instances ot extraordinary size of a limb, a width of three and a half, or even four inches, and two or three feet of additional length may be desirable. In cases where the leg is very slender, the length I mention will be more than is needed, but the superfluous bandage may be wound round the leg under the knee, or, of course, cut off to suit the exact requirements of the case.

I need not relate how I gradually came to the conclusion that such a bandage as I have described, *without any other means or appliance whatever*, is all that is necessary for the perfect and *permanent* cure of all curable non-specific ulcers of the leg. Such is the fact, and I have no hesitation whatever in asserting that all former other methods of treating such ulcers may and should be abandoned entirely, no matter how illustrious their authors. I dare say such an assertion from an obscure private practitioner may appear presumptuous, but it is very deliberately made. For twenty-five years I have been constantly observing cases of every variety of ulcer of the leg under treatment by the strong elastic bandage. During those years, I have had ample opportunity to observe the results of other methods of treatment ; and before I fully adopted my present practice, had frequent and full personal experience of the infinite trouble to the surgeon, tediousness of duration of treatment, and insecurity and want of permanence of result from the treatment of such cases by Baynton's and other familiar methods. I am fully aware of the importance and value of the means I advocate, and very solicitous that the profession should, at any rate, test the truth of what I assert by the practical use of the bandage. I wish that the announcement of this method of treatment might have the advantage of coming from one of those recognized authorities and magnates of the profession, who have forced so many worthless methods into practice, simply because they were *their* methods. "Truth is great and *must* prevail." It is a sweet consolation to believe this axiom, but the highest and best truths, coming from obscure and unknown sources, are very apt to be disregarded, while error and fallacy, backed by a mighty name, are eagerly hailed and adopted. The method I advocate is so easily tested, a single case of ulcer of the leg carefully observed will demonstrate its advantages clearly and perfectly, and as people with uncured ulcers of the leg abound everywhere, I cannot help hoping that, although I am unknown in every way as an authority, the method will be fairly tried, and win, by its intrinsic and evident merit, a permanent place in surgical practice.

The bandage is to be applied by taking one turn just above the ankle, then one over the instep, round the sole of the foot, then round the ankle and, spirally, up over the leg, to the knee, at which point what remains unapplied should be wound round the limb, and the tapes firmly tied. Each turn should overlap that before it from $\frac{1}{2}$ to $\frac{3}{4}$ of an inch. No skill whatever is requisite, as the bandage is simply carried round and round, without any of the nice reduplication which is necessary for the proper and useful application of the ordinary bandage. The best time to apply it is the first thing in the morning, before the veins of the leg have become distended from the impeded column of blood. The very best way is for the patient to apply it in bed, before assuming the upright position. If, in these circumstances, the bandage is applied with just enough snugness not to slip down, it will, at once, on the patient standing up, become of exactly the right degree of closeness of application. This is all that need be said in regard to the application of the bandage. Thus applied, it will remain unmoved during the whole day, no matter how active and continual the exercise or labor of the patient. A theoretical objection to be met is that that portion of the foot below the bandage must become œdematous. Such certainly would be the effect, of an ordinary bandage, applied with sufficient tightness to be of any use, but the fact is, that no œdema follows the proper use of the rubber bandage. Indeed, in some cases of ulcer of the leg, a certain degree of œdema of the foot is found, due to the weakened and distended veins, and consequent impediment to the circulation. This œdema is rapidly removed by the use of the strong elastic bandage. These facts are illustrative of the reasons why its application alone is so entirely sufficient for the best possible treatment of the varicose ulcer. That sort of ulcer and the œdema often accompanying it, are due to the too great quantity of blood in the limb. The bandage expels the superfluous blood, and while it supports the weakened walls of the distended veins, does not stop, but really facilitates the circulation through them. The pressure exercised by the bandage is a very efficient means towards the absorption of interstitial deposits, serous or fibrinous, effused among the tissues of ulcerated legs.

The patient wears the bandage all day. At bed-time it is to be removed. When this is done, its inner surface and the skin, which it had covered, will be found bathed in profuse sweat; this is to be wiped from the limb with a dry cloth. A bit or bits of thin, soft, old linen, moistened with castor or sweet oil, is to be laid on the ulcerated spots, and kept in place by a few turns of cotton roller bandage. This is the treatment, with recumbent position all night, and the bandage and the erect position all day. The bandage should be wiped with a wet sponge, and hung up to dry, or, of course, it may be wiped dry at once, and rolled up, with the tapes inside, for use on the following morning. This is the whole treatment I have employed for the permanent and solid cure of many hundreds of ulcerated legs, during the past twenty-five years. Now and then, a peculiarly diseased condition of the skin has led me to recommend a daily or less frequent washing with Packer's tar soap[1], and occasionally a carbolized, or other solution where there was an intensely itching condition of the surface. As I am writing for physicians, I need not enter into details of treatment of exceptional cases. What is required by the peculiar complications of certain cases will suggest itself to the intelligent practitioner. For ulcer of the leg, *uncomplicated* by other disease of the skin, the bandage alone is all that is needed.

During the first two or even three weeks that the bandage is used, a more or less numerous crop of papules will appear on the skin to which it is applied. These run, rapidly, into suppuration, discharge their contents and disappear. Generally they are small, but occasionally larger, and now and then of the character of small boils. Whatever their size or number, the best possible treatment for their resolution is by the bandage. This is practically the best possible poultice. In a wonderfully short time these little pustules run their course in the warm moist atmosphere under the bandage. Each of these pimples or pustules represents an obstructed follicle or duct. After they have ceased to appear, the skin becomes and remains entirely unobstructed, no matter how long the bandage is worn. A few patients cannot, or *think* they cannot bear the rubber next the skin. In many hundreds of cases, I have met but three or four in whom there was a want of perfect and easy tolerance. In these exceptional cases I was obliged to wind the limb with a linen bandage, over which I applied the rubber. Only one of these exceptional cases was of ulcer of the leg; the others were cases in which the bandage was applied for disease or result of injury of the knee joint. I have said that the strong elastic bandage *alone* is all that is needed for the successful treatment of all non-specific

1. I have found "tar soap," which contains a large amount of tar, properly saponified, of very great value in certain diseased conditions of the skin, and rectal and vaginal mucous membranes. A great deal, however of so-called tar soap is so merely in name, and intended for toilet use. I formerly used the Hamburg Thev Seife, which answers very well, but is absurdly expensive. For two or three years, I have made use exclusively of Packer's tar soap, an American product, far superior to even that from Hamburg, and sold at a very reasonable price.

ulcers of the lower extremity. I repeat it with the utmost confidence based on a very large and long experience. One and the chief reason that I have so long forborne to communicate to the profession a method which I consider so valuable was that I wished to accumulate such a number and variety of cases in which it had been successfully employed as would leave no room for doubt, and, being quite without the vast facilities afforded by hospital practice, such accumulation was a gradual process.[1] The form of ulcer which yields most readily; with a rapidity which is sometimes really wonderful, is that most common of all, the varicose ulcer. The reasons for this are obvious. That sort of ulcer is caused and maintained by malnutrition of the skin from the engorged, impeded circulation, which is at once relieved by the bandage. In those cases caused by a languor and imperfection of circulation in the legs, from deficiency in quality or quantity of blood, or feebleness of the heart's action, the bandage accomplishes a cure by the warmth and moisture it secures, favoring the circulation in the cutaneous capillaries and inducing a *removal* of blood to the surface. In these cases, familiar to all surgeons for their obstinate resistance to treatment and the imperfection and unreliability of their cicatrization, the ulcers called by Hippocrates, and the classic surgeons "Chironian"[2]—round or

1. During most of this time, although I have spoken very freely to physicians of my use of these bandages and furnished them with opportunity to test their usefulness, and, in this way, the method has become widely known, I have never hitherto published any of the results of my experience. I have never, in a pretty extensive reading of periodical medical literature, met with any account of the use of the strong elastic bandage in the way I recommend. In the London *Practitioner* for May, 1876, there is a very brief paper entitled "Use of blisters in chronic ulcers," by S. D. Turney, of Circleville, Ohio. It has nothing to do with blisters, but *is* a very interesting record of the very rapid cure of five or six bad ulcerated legs by applying Esmarch's bandage every day as tightly as could be borne by the patient, for a very *short* time. So far as I know, the paper has attracted no notice or comment, but it notes an important fact, and I refer the reader to it, as, to a certain extent, corroborative of what I have written.

2. *Chironian Ulcer*, So called because so difficult of cure that Chiron (who taught Apollo fiddling and physic) alone, or one his equal, might cure it.

roundish, with perpendicular sides, as if punched out of the whole thickness of the much-thickened skin, with hard, white, scathing, often almost cartilaginous edges, yield to the bandage and to that alone, and with far more perfect and stable results than by other methods of treatment, but they are cured much more slowly than any other variety of non-specific ulcer. Before anything like *reliable* cicatrization of these ulcers can occur, the hard edges must be entirely got rid of. The constant pressure of the strong elastic bandage is an efficient agent in promoting the absorption of this impediment to cure; but it is a slow process. In such cases I recommend that the patient should wear the bandage night as well as day, while in the very large class of ulcers caused and maintained by a varicose condition of the veins, I direct the bandage to be worn only during the day, as before stated.

In, perhaps, the worst *curable* case I ever saw of this inveterate sort of ulcer, in an old, feeble, ill-nurtured patient who had been, off and on, under treatment for nearly nine years, whose ulcer had been nominally "cured" again and again, and in each instance, almost immediately on resuming labor, the cicatrix had broken down, I used the bandage alone as a test case. Of course I could have much expedited the cure by removing the gristly border of the ulcer by caustic, or the knife, but I depended on the bandage only, and in four months, during which the patient continued to labor without any intermission, his ulcer was solidly and well healed, and has now, for nearly five years, remained so. I may say here that not only with this method is the patient *allowed* to continue his ordinary avocations, however laborious, but is much better able to work while wearing the bandage than he would be without it. This is particularly to be noticed in all varicose conditions of the leg. I have had many cases in which it was only by wearing such a bandage that a patient could do his daily work. I shall refer to this point again in a future paper, in which I hope to demonstrate the extreme value of these bandages as a palliative in cases of varicose veins of the leg, *uncomplicated* with ulcer. I am aware how hastily and roughly this paper has been thrown together, but I believe it indicates pretty clearly my method of treating ulcers by the strong elastic bandages, and my perfect confidence in that method, and that is its entire end and object. If it attracts the notice of the profession, and leads to a practical approving of a method so easily tested, I shall have accomplished what I wished and intended. In another paper, prepared at the request of the publication committee of the American Medical Association, I have described six cases illustrative of results of treatment of a variety of ulcers, and other diseases and injuries. I suppose that paper may be published in due time, and to it I refer the reader. I would also say that I shall be very happy to answer any inquiry as to points connected with the subject on which my readers may wish to be informed.

27 Dudley street, Boston, October 1st, 1877.

*Postscript.*—Since my return from Chicago, and as a result of my remarks at the meeting there, I have received very numerous applications for bandages, and for the address of a dealer from whom they could be obtained. To meet the requirements of the profession I have made arrangements with Messrs. T. Metcalf & Co., 39 Tremont St., and Messrs. Leach & Greene, 1 Hamilton Place, Boston, who will, in future, have always on hand an ample supply of bandages for the leg, made under my direction and inspected by myself. These gentlemen will also keep on hand bandages of the exceptional length and breadth I have alluded to, and also the bandaging of which any required number of feet may be obtained for the very varying requirements for the different joints.

I find, on reviewing what I have written, that I have omitted to state that, after cure of varicose ulcer, many of my patients, whose occupations require much standing or walking, continue to wear the bandage, both for the relief of their varicose veins and as a preventive of a return of the ulcer. Other patients I recommend to resume the use of the bandage if there is any symptom of return of the ulcer, or if they are obliged to be much on foot.

